# Reliability of Fracture Classifications in Surgically Treated Simple Ankle Fractures

**DOI:** 10.7759/cureus.115556

**Published:** 2026-08-31

**Authors:** Rasmus P Severinsen, Lise Hellegaard, Jacob S Engbjerg, Jacob Venborg, Frederik L Hansen, Hans-Christen Husum, Daniel Wæver

**Affiliations:** 1 Department of Orthopedics, Regional Hospital Randers, Randers, DNK; 2 Department of Radiology, Regional Hospital Randers, Randers, DNK; 3 Department of Orthopedics, Aarhus University Hospital, Aarhus, DNK

**Keywords:** ankle fractures, classification system, interobserver reliability, intraobserver agreement, orthopedic surgeries

## Abstract

Background

Reliable fracture classification is essential for communication, treatment planning, and research. Several systems classify ankle fractures, but their reproducibility remains debated. We evaluated inter- and intrarater reliability and agreement of the Weber, Lauge-Hansen, and AO/OTA classifications among orthopedic and radiology clinicians.

Methods

We retrospectively identified 265 surgically treated ankle fractures at Randers Regional Hospital, Denmark, from February 2020 to December 2024. Four observers (orthopedic resident, orthopedic consultant, radiology intern, radiology resident) independently classified preoperative radiographs. After three months, they reassessed 50 randomly selected cases. Reliability was quantified using Gwet's agreement coefficient (AC) with 95% confidence intervals and percent agreement.

Results

The Weber classification demonstrated very good interrater reliability (AC 0.90; 91% agreement). Lauge-Hansen (AC 0.70; 72%) and AO/OTA (AC 0.66; 69%) showed good agreement. Intrarater reliability was highest for Weber across all observers (AC 0.82-0.89).

Conclusion

The Weber classification provides superior reliability compared with Lauge-Hansen and AO/OTA when applied to plain radiographs of surgically treated ankle fractures.

## Introduction

Ankle fractures are common trauma-related injuries with an incidence rate of 157-179 per 100,000 patient years, representing nearly 10% of all reported fractures [1]. Incidence follows a bimodal distribution with an early peak in young men and a late peak amongst elderly women. The latter is becoming larger due to an increasingly elderly population [1].

Ankle fractures range from simple extra-articular injuries to complex intra-articular fracture patterns. The distal fibula fracture, or lateral malleolus, is the most common ankle fracture, representing more than half of all cases [1].

A fracture classification system must be reproducible. It should also entail information regarding the severity of the given fracture and the prognosis of the injury. Preferably, it can act as a tool to guide the choice of treatment [2-4].

Traditionally, the Weber classification [5], the expanded AO-OTA classification [6], and the Lauge-Hansen classification [7] have been used to classify ankle fractures based on preoperative radiographs. The Weber is anatomically founded with the fibular fracture level relative to the syndesmosis, while the Lauge-Hansen classification is based on injury mechanism and foot positioning. These two classifications remain the most widely used, whereas AO-OTA is a more detailed, morphology-based classification. The simpler Weber classification has shown superior reproducibility [8], although trimalleolar fractures appear to be reliably classified with the AO-OTA classification [9].

Despite widespread use of ankle fracture classification systems, their clinical applicability may be limited by inconsistent reproducibility. Previous studies have reported mixed interrater reproducibility of the AO and Lauge-Hansen classifications, ranging from fair to very good [8-12]. However, few studies have evaluated these classification systems simultaneously across clinically relevant specialties and included intrarater reliability. Therefore, the comparative reliability of commonly used systems in a real-world multidisciplinary setting remains uncertain.

We aimed to assess inter- and intrarater reliability of the Weber, Lauge-Hansen, and AO/OTA classifications among orthopedic surgeons and radiologists of varying experience from pre-operative radiographic imaging of surgically treated ankle fractures using Gwet's AC and Percent Agreement calculations.

## Materials and methods

The manuscript was written according to the guidelines for reporting reliability and agreement studies (GRRAS) [13].

Study population

The study population was based on a cohort established in an unpublished work assessing the post-operative complications for surgically treated simple malleolar fractures. The cohort consisted of patients who had undergone ankle fracture surgery at Randers Regional Hospital, Denmark, from February 2020 through December 2024. Eligible cases were identified using procedure codes (KNHJ40-90-3) combined with diagnostic codes (DS826, DS827A, DS827B) from the Business-Intelligence portal in the Central Denmark Region, Denmark.

The following cases were excluded: patients with less than one-year follow-up, duplicates, open fractures, primary external fixation, concomitant significant injuries, faulty diagnosis, lack of imaging, and osteosynthesis techniques other than standard lateral plating with supplementary screws (i.e., fibula rods, retrograde nailing, K-wires, cerclage, medial or posterior plating, Tight-Rope (Arthrex, Naples, Florida, USA))

Imaging

Radiographic imaging was obtained through the Picture Archiving and Communication System (PACS). Imaging consisted of three standard preoperative ankle radiographic projections (anterior-posterior, lateral, and oblique) prior to surgery. 

Observers and assessment

Preoperative imaging from eligible cases was reviewed and classified according to Weber, Lauge-Hansen, and AO/OTA classifications by four observers. After a three-month period, reassessment of 50 randomly selected cases was performed to evaluate intra-observer reliability. Written descriptions and visual images of the classification system were used during assessment. The assessment was performed individually and independently without access to other observer's assessments or thier own prior assessment. There were no standardized training or calibration prior to assesment. The observer panel consisted of four medical doctors: an orthopedic trauma consultant, an orthopedic senior resident, a radiology intern, and a radiology resident.

Statistical analysis

Prior to data analysis, data were collected in preformatted forms using a REDCap database (Vanderbilt University, Nashville, Tennessee, USA). Statistical analyses were performed in Stata 18 (StataCorp LLC, College Station, Texas, USA).

Reliability was quantified using Gwet's AC1 with 95% confidence intervals and percent agreement. The agreement coefficient was unweighted, as the fracture classification categories were considered nominal rather than ordinal. Confidence intervals were calculated using the variance estimation procedure implemented in Stata. Cohen's kappa was initially calculated; however, despite high observed percent agreement, low kappa values were observed across all classification systems. Given the low variance of some classification categories and the resulting skewed distribution of observations, Gwet's AC1 was subsequently used to provide a more stable estimate of agreement and avoid the underestimation of agreement associated with Cohen's kappa, a phenomenon commonly referred to as the kappa paradox [14-16].

Interrater reliability across all included fractures was assessed for the three fracture classification systems using ratings from all four observers simultaneously. Intrarater reliability was assessed from an arbitrary subset of 50 ankle radiographs from the cohort. There was no further randomization or stratification applied to this subset. There were no missing or unclassifiable ratings in the dataset.

To interpret results, Gwet’s AC was applied to Altman et al.'s [17] scale for interpretation of kappa-values (Table 1). 

**Table 1:** Scale for Gwet's AC interpretation Gwets AC ranges with corresponding agreement brackets, modified from Kappa suggestions by Altmann et al. [17]

Gwet's AC	Agreement
≤0.20	Poor
0.21-0.40	Fair
0.41-0.60	Moderate
0.61-0.80	Good
0.81-1.00	Very good

## Results

We identified a total of 384 patients, of which 265 were included as our study population. Three further patients from the originally established cohort were excluded due to insufficient imaging upon review (Table 2). Fifty subjects were reassessed by observers. All four observers (orthopedic resident, orthopedic consultant, radiology intern, and radiology resident) completed independent primary assessment for all subjects and 50 reassessments as intended.

**Table 2:** Inclusion and exclusion Inclusion and exclusion with identified number of patients, n. Three patients met two exclusion criteria, resulting in 122 exclusion criteria in 119 excluded patients.

Inclusion criteria	n
Surgically treated ankle fracture at Randers Regional Hospital between February 2020 and December 2024 in adults, age =/> 18	384
Exclusion criteria	n
Duplicate records	7
Death before 12-month follow-up	3
Open fracture	2
Initial external fixation	6
Significant concomitant injury	3
Other osteosynthesis modality than semitubulary plate and screws	65
Foreign citizens or individuals with residence outside the hospital's region	27
Incomplete imaging	3
Faulty registration	6

Demographics

Women represented 62.3% of the cohort (n=167). The mean age at time of surgery was 57.9 years, the mean body mass index (BMI) was 28.4 kg/m2, and 217 patients (81%) were either classified as American Society of Anesthesiologists Physical Status Classification (ASA) 1 or 2. 

Interrater reliability 

As shown in Table 3, the Weber classification demonstrated the highest level of interrater reliability with a Gwet's AC of 0.90 and a percent agreement of 91%, indicating very good agreement among raters. The AO/OTA classification showed good agreement with a Gwet's AC of 0.66 and a percent agreement of 69%. Similarly, the Lauge-Hansen classification demonstrated good agreement with a Gwet's AC of 0.70 and a percent agreement of 72%.

**Table 3:** Interrater reliability and agreement of the Weber, Lauge-Hansen, and AO/OTA classification systems Percent Agreement and Gwet's AC values with (95% CI), n=265

Weber	
Percent agreement	91 (89-94)
Gwet's AC	0.90 (0.87-0.93)
Lauge-Hansen	
Percent agreement	72 (69-76)
Gwet's AC	0.70 (0.66-0.74)
AO/OTA	
Percent agreement	69 (65-73)
Gwet's AC	0.66 (0.62-0.70)

Intrarater reliability

As shown in Table 4, overall reliability ranged from good to almost perfect across the Lauge-Hansen, AO/OTA, and Weber classification systems. Point estimates suggested superior intrarater reliability for the Weber classification compared with Lauge-Hansen and AO/OTA systems.

**Table 4:** Intrarater reliability of the Weber, Lauge-Hansen, and AO/OTA Classification Systems reassessed at three months Percent Agreement and Gwet's AC values with (95% CI), n=50

	Orthopedic resident	Orthopedic consultant	Radiology intern	Radiology resident
Weber				
Percent agreement	92 (84-100)	90 (81-99)	88 (79-97)	88 (79-97)
Gwet's AC	0.89 (0.77-1)	0.83 (0.68-0.99)	0.84 (0.69-0.98)	0.82 (0.67-0.97)
Lauge-Hansen				
Percent agreement	74 (61-87)	72 (59-85)	62 (48-76)	74 (61-87)
Gwet's AC	0.70 (0.55-0.85)	0.68 (0.53-0.83)	0.57 (0.41-0.73)	0.70 (0.56-0.85)
AO/OTA				
Percent agreement	78 (66-90)	82 (71-93)	64 (50-78)	66 (52-80)
Gwet's AC	0.73 (0.59-0.88)	0.78 (0.64-0.92)	0.59 (0.42-0.75)	0.60 (0.43-0.76)

For the orthopedic consultant, consistently high intrarater reliability with Gwet's AC values ranging from good to almost perfect across all three classifications, and a high percent agreement was observed. For the orthopedic resident, overall good intrarater reliability was observed, with the highest observed agreement for the Weber classification. 

For the radiology resident, good to very good reliability across classification systems was observed. For the radiology intern, more variable performance was observed with lower agreement for Lauge-Hansen and AO/OTA classifications, but higher reliability for Weber. Across all observers, the Weber classification consistently yielded the highest intra-observer reliability, while Lauge-Hansen and AO/OTA classifications showed lower agreement, ranging from moderate to good.

## Discussion

In this study of 265 surgically treated ankle fractures, we assessed inter- and intrarater reliability of the Weber, Lauge-Hansen, and AO/OTA classification systems across orthopedic and radiology clinicians with varying levels of experience. Overall, good to very good reliability was observed across all three systems. The Weber classification demonstrated the highest reproducibility, whereas the AO/OTA classification showed the lowest level of agreement. 

A key finding of this study is the superior reliability of the Weber classification. This likely reflects its structural simplicity, consisting of only three categories based on fibular fracture level, which is typically readily identifiable on standard radiographs. In contrast, the Lauge-Hansen classification requires inference of injury mechanism, introducing a subjective component, while the AO/OTA classification involves detailed morphological distinctions that increase interpretive complexity. These inherent differences likely explain the observed gradient in agreement, where increasing classification complexity corresponded with decreasing reproducibility. This observation supports the notion that simpler classification systems may yield more consistent use in clinical practice and align with the broader literature [2,18].

Our findings are in accordance with previous studies reporting higher reproducibility of the Weber classification compared with more complex systems. Viberg et al. [10] and Delaney et al. [8] demonstrated similar results to ours with moderate to very good interobserver agreement for stability-based ankle fracture classifications, in contrast to Fonseca et al. [11] and Alexandropoulos et al. [12] showing only fair to moderate reproducibility of the AO/OTA and Lauge Hansen classifications, highlighting the ambiguity of the existing literature. 

Previous studies on the topic used only orthopedic specialists or orthopedics in training, while we included radiologists. Both orthopedic and radiology observed generally achieved moderate to very good agreement. Importantly, by applying Gwet's agreement coefficient, the present study addresses limitations associated with the kappa paradox and may provide a more robust estimate of agreement in datasets with skewed category distributions [14-16].

We observed a trend towards higher intrarater reliability and higher consistency across classification systems for more experienced clinicians. This suggests that familiarity with fracture patterns and greater exposure to radiographic interpretation may improve reproducibility. However, the literature reports contrasting results on this [10,19-20]. 

In our study, however, even among experienced observers, lower agreement persisted for the Lauge-Hansen and AO/OTA classifications compared with Weber. This indicates that complexity within classification systems cannot be fully compensated for by clinical experience alone and may represent an intrinsic limitation of these systems.

These findings have important clinical implications. The high reliability of the Weber classification supports its use in routine practice, whereas the lower agreement for the AO/OTA and Lauge-Hansen classifications may limit their applicability based on plain radiographs alone. While more complex systems may provide greater morphological detail, their clinical value depends on whether this translates into improved prognostic or treatment-related decision-making, which is still controversial [3,21]. Classification systems should therefore balance detail with reproducibility to optimize clinical utility.

Limitations

Several limitations of this study should be considered. First, a substantial proportion of radiographs were obtained after fracture reduction and casting due to local clinical guidelines on dislocated ankle fractures, possibly obscuring anatomical detail and influencing image interpretation due to blurring from casting and bandage material, which, subsequently, may have influenced classification agreement and reproducibility.

The study population consisted exclusively of surgically treated fractures, resulting in a selection towards more severe and unstable injury patterns. Stable fractures, including Weber type A and AO/OTA type A injuries, were excluded, limiting generalizability to non-operative settings. 

The study was conducted at a single institution with four observers, which may affect external validity despite the inclusion of both orthopedic and radiology clinicians with varying experience levels. The study was not designed nor powered to establish equivalence or statistically compare reliability between specialties. Further multicentre studies with more observers are required before generalizing these findings across specialties, experience levels, and fracture populations.

Finally, while Gwet's AC provides advantages over Cohen's kappa in the presence of skewed distributions, comparisons with existing literature using kappa statistics should be interpreted with caution.

## Conclusions

In this single-centre cohort of surgically treated ankle fractures, the Weber classification demonstrated higher observed interrater and intrarater agreement than the Lauge-Hansen and AO/OTA systems when applied to plain radiographs of ankle fractures requiring surgical intervention. These findings suggest that the Weber system may be more reproducible in routine radiographic assessment; however, larger multicentre studies involving more observers and the full spectrum of operative and non-operative ankle fractures are required.
